# Supplementary material for: Use of hiPSC-Derived Cardiomyocytes to Rule Out Proarrhythmic Effects of Drugs: The Case of Hydroxychloroquine in COVID-19
Source: Front Physiol. 2022 Jan 27;12:730127. doi: 10.3389/fphys.2021.730127 (PMC8829511; doi:10.3389/fphys.2021.730127)
Supplement: Supplementary file 1 [file Data_Sheet_1.pdf]

Supplementary Material

Table S1
List of reagents used in the study.

Reagent	Company	Product code
**Vitronectin (VTN-N) Recombinant Human Protein, Truncated**	Thermo Fisher Scientific [GIBCO]	A14700
**Matrigel® Corning® hESC-Qualified Matrix, LDEV-free**	Corning	354277
**Fibronectin bovine plasma**	Merck [Sigma-Aldrich]	F1141
**Essential 8™ Flex Medium Kit**	Thermo Fisher Scientific [GIBCO]	A2858501
**RPMI 1640 w/ L-Glutamine**	Euroclone	ECB2000
**RPMI 1640 Medium, no glucose**	Thermo Fisher Scientific [GIBCO]	11879020
**DMEM/F-12**	Thermo Fisher Scientific [GIBCO]	11320033
**B-27™ Supplement**	Thermo Fisher Scientific [GIBCO]	17504044
**B-27™ Supplement, minus insulin**	Thermo Fisher Scientific [GIBCO]	A1895601
**DPBS, no calcium, no magnesium**	Thermo Fisher Scientific [GIBCO]	14190094
**EDTA (0.5 M), pH 8.0, RNase-free**	Thermo Fisher Scientific [Invitrogen]	AM9262
**TrypLE™ Select Enzyme**	Thermo Fisher Scientific [GIBCO]	A1217701
**RevitaCell™ Supplement**	Thermo Fisher Scientific [GIBCO]	A2644501
**CryoStor® CS10**	StemCell Technologies	7930
**CHIR-99021 HCl**	Selleckchem	S2924
**IWR-1**	Merck [Sigma-Aldrich]	I0161
**24-well Plate with Gold Electrodes on FR4**	Multichannel Systems	24W700/100F-288
**96-well Plate with Gold Electrodes on FR4**	Multichannel Systems	96W700/100F-288
		i
**Hydroxychloroquine Sulfate**	Selleckchem	S4430

Table S2
Baseline MEA parameters for all the lines used in the study.

Cell Line	Mean FPD (ms)	FPD sem (ms)	Mean RR (ms)	RR sem (ms)	FPD-RR Fitting Coefficient Pr(>|t|)	Mean cFPD (ms)	cFPD sem (ms)	cFPD-RR Fitting Coefficient Pr(>|t|)	N of MEAs
**WT**	500.4	15	1639.8	50.5	9.72e-05	393.9	9.9	0.729	58
**WT2**	286.1	5.1	890.5	12.3	0.875	304.1	5.7	0.0314	48

**LQT1**	174.9	4.9	599.6	17.3	2.14e-12	227.5	5.0	0.193	106
**JLNS**	225.5	13.6	546.4	37.1	<2e-16	303.9	11.7	0.000203	62
**CALM-LQTS**	308.5	17.7	1359.9	62.6	4.31e-05	263.7	13.1	0.0864	56

Patch Clamp

hiPSC-CMs were dissociated with TrypLE Select 10X (Thermo Fisher Scientific) and plated sparsely on Matrigel-coated glass coverslips (10 mm Ø). Isolated hiPSC-CMs were patched 3-10 days after dissociation. Tyrode's solution contained (mM): NaCl 154, KCl 5.4, $CaCl_2$ 1.8, HEPES-NaOH 5, D-Glucose 5.5. pH was set to 7.35 with NaOH. Intracellular solution contained (mM): K-Aspartate 125, KCl 20, NaCl 10, Na_2-ATP 5, HEPES 10. pH was set to 7.3 with KOH. Amphotericin B 0.22 mM in DMSO was added to the intracellular solution to record APs.

APs were recorded with a Molecular Devices digidata 1440A and a Molecular Devices Axopatch 200B amplifier at physiological temperature (~37 °C). No holding current injection was used to hyperpolarize the resting membrane potential (E_{diast}).

The duration and amplitude of the current pulses used to elicit the APs were in a range of 2-3 ms and 0.5-1.5 nA. Signals were digitized at 5 kHz and filtered at 2 kHz with a low pass Bessel filter.

Liquid junction potential was calculated according to the stationary Nernst-Planck equation using LJPcalc (Harden, SW and Brogioli, D (2020). LJPcalc [Online]. Available: https://swharden.com/software/LJPcalc, Accessed on 03/08/2021). The calculated LJP was 13.922 mV. The measured LJP was -11.2 +- 0.9 mV. Patch clamp data were obtained from multiple independent differentiations for each line.

Supplementary Figures

Figure S1

A) Percentage of hiPSC-CMs which had measurable AP parameters after 5 minutes in 10 µM HCQ during 1 Hz pacing (grey) against those which were completely depolarized and could not be further stimulated (red).
B) Average AP data from WT2, JLNS and CALM-LQTS at baseline (grey) and after acute stimulation with 10 µM HCQ. N = 16, 22, 9. * indicates $p < 0.05$ vs Baseline.
C) Representative AP traces (top), magnification of phase 0 (middle) and first derivative of the middle panel (bottom) from the WT2 (left), the the JLNS line (middle) and the CALM-LQTS line (right) at baseline (black) and after acute stimulation with 10 µM HCQ (red).

Figure S2

Time-course of an isolated hiPSC-CM from the JLNS line paced at 1 Hz during 10 µM HCQ exposure. The insets indicate pre-exposure (left), EAD and arrhythmias during exposure (middle) and washout (right).

Figure S3

A) Raw data for HCQ effect on FPD.
B) Raw data for HCQ effect on RR.
C) Raw data for HCQ effect on cFPD. In each plot, * indicates $p < 0.05$ vs Baseline.

Figure S4

A) Arrhythmogenic events detected in JLNS hiPSC-CMs.
B) Arrhythmogenic events detected in CALM-LQTS hiPSC-CMs.
