# Supplementary figures and images for: Use of hiPSC-Derived Cardiomyocytes to Rule Out Proarrhythmic Effects of Drugs: The Case of Hydroxychloroquine in COVID-19
Source: Front Physiol. 2022 Jan 27;12:730127. doi: 10.3389/fphys.2021.730127 (PMC8829511; doi:10.3389/fphys.2021.730127)

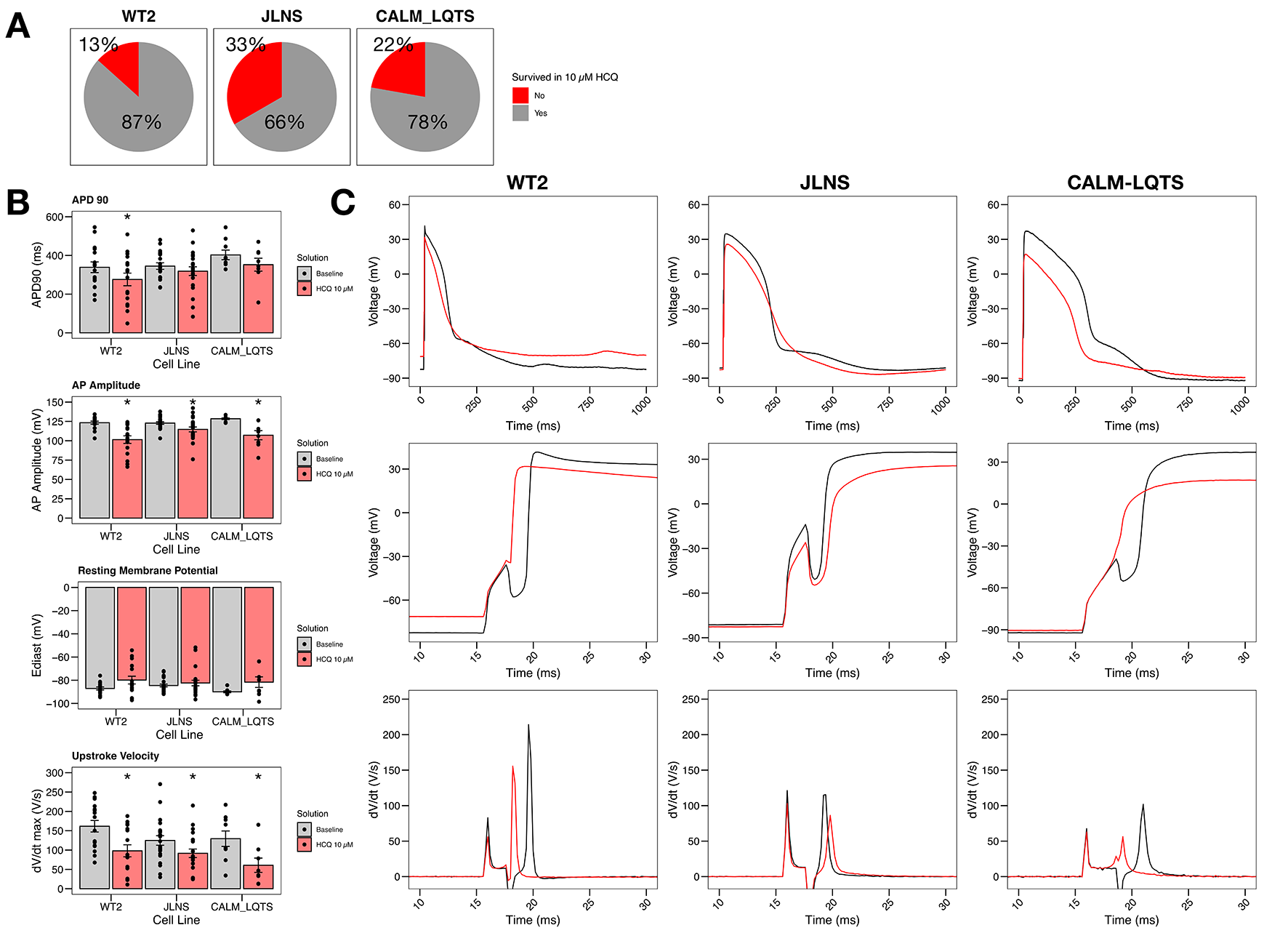

Supplement: Supplementary file 2 [file Image_1.TIFF]

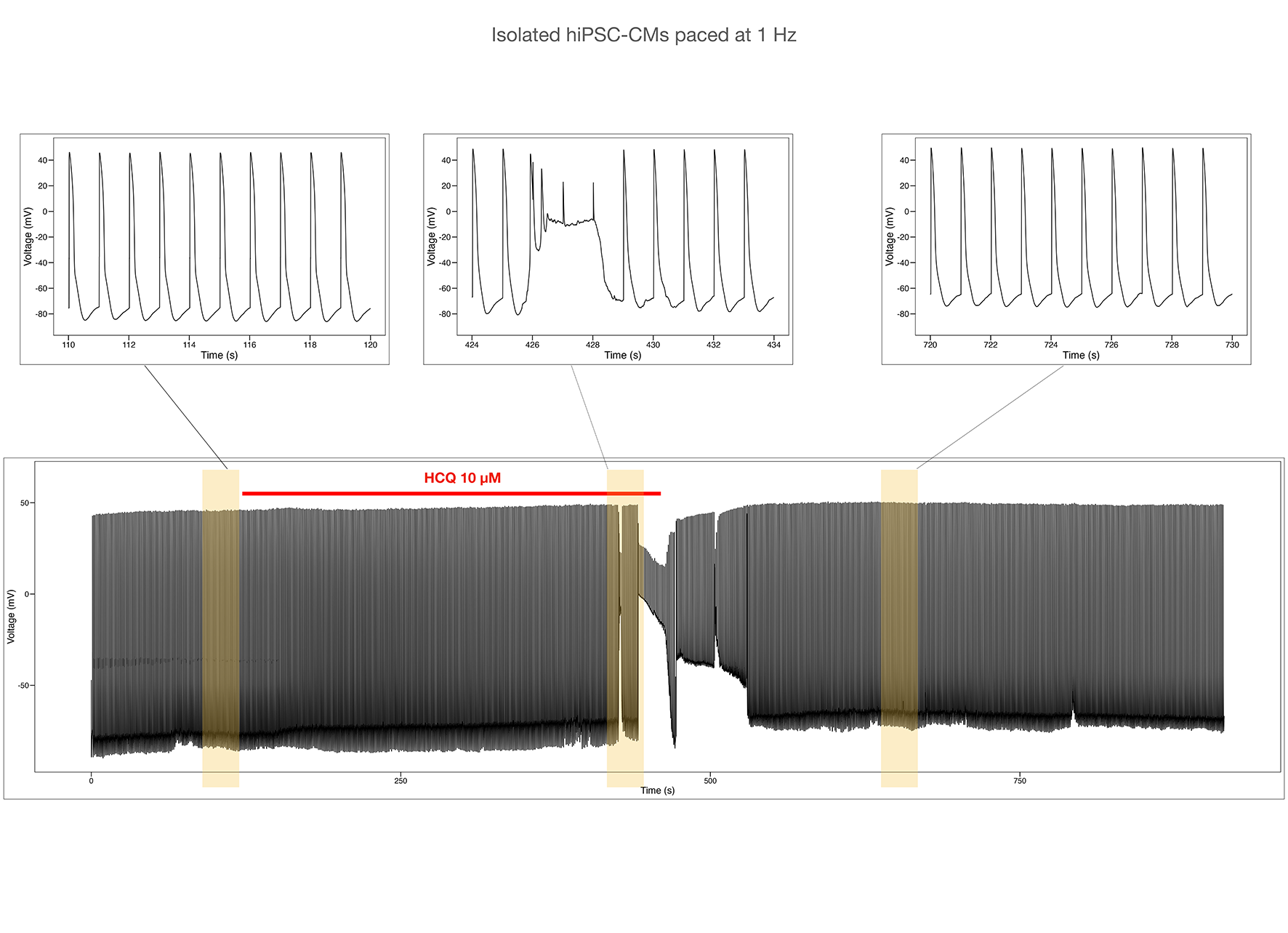

Supplement: Supplementary file 3 [file Image_2.TIFF]

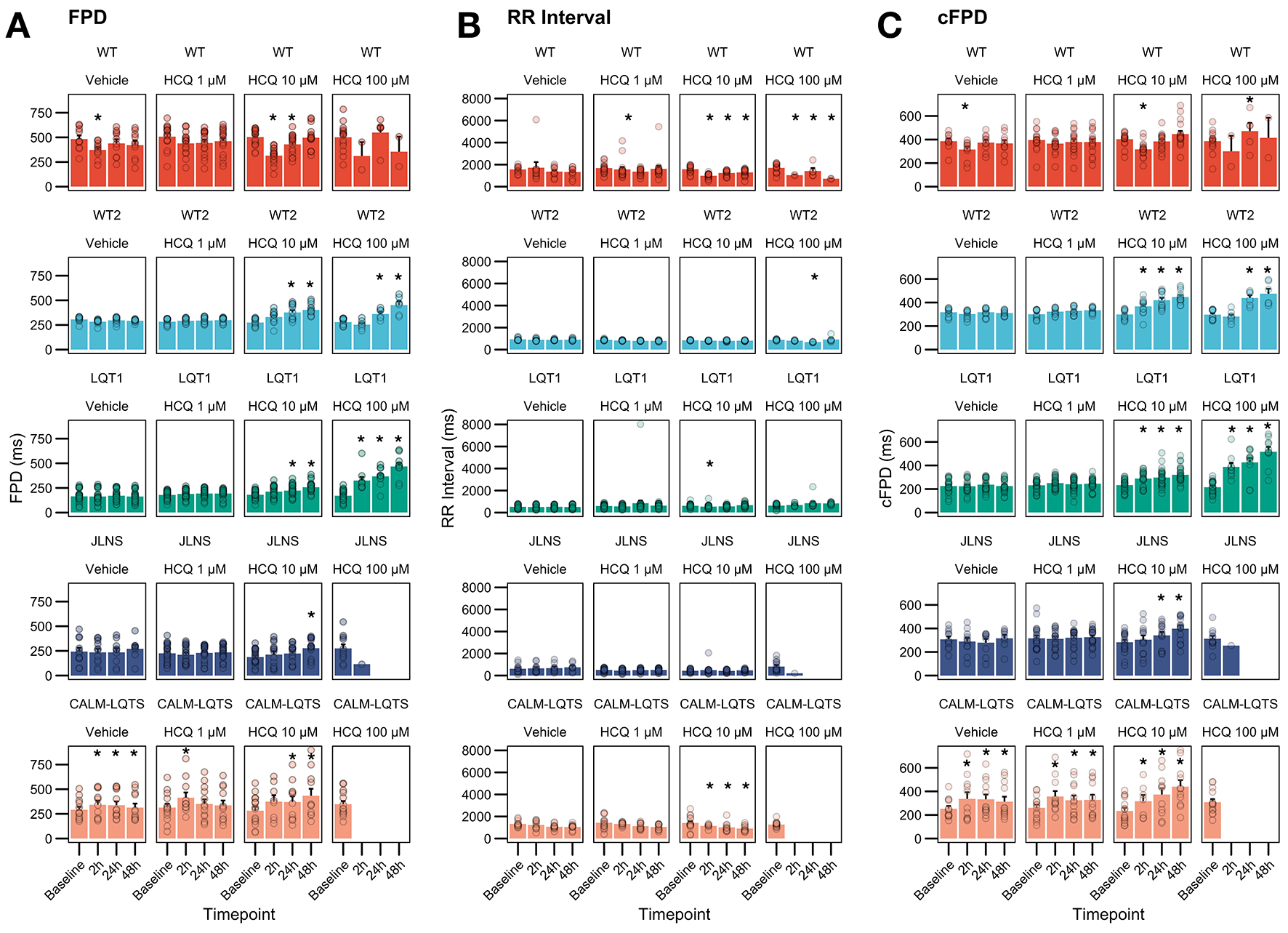

Supplement: Supplementary file 4 [file Image_3.TIFF]

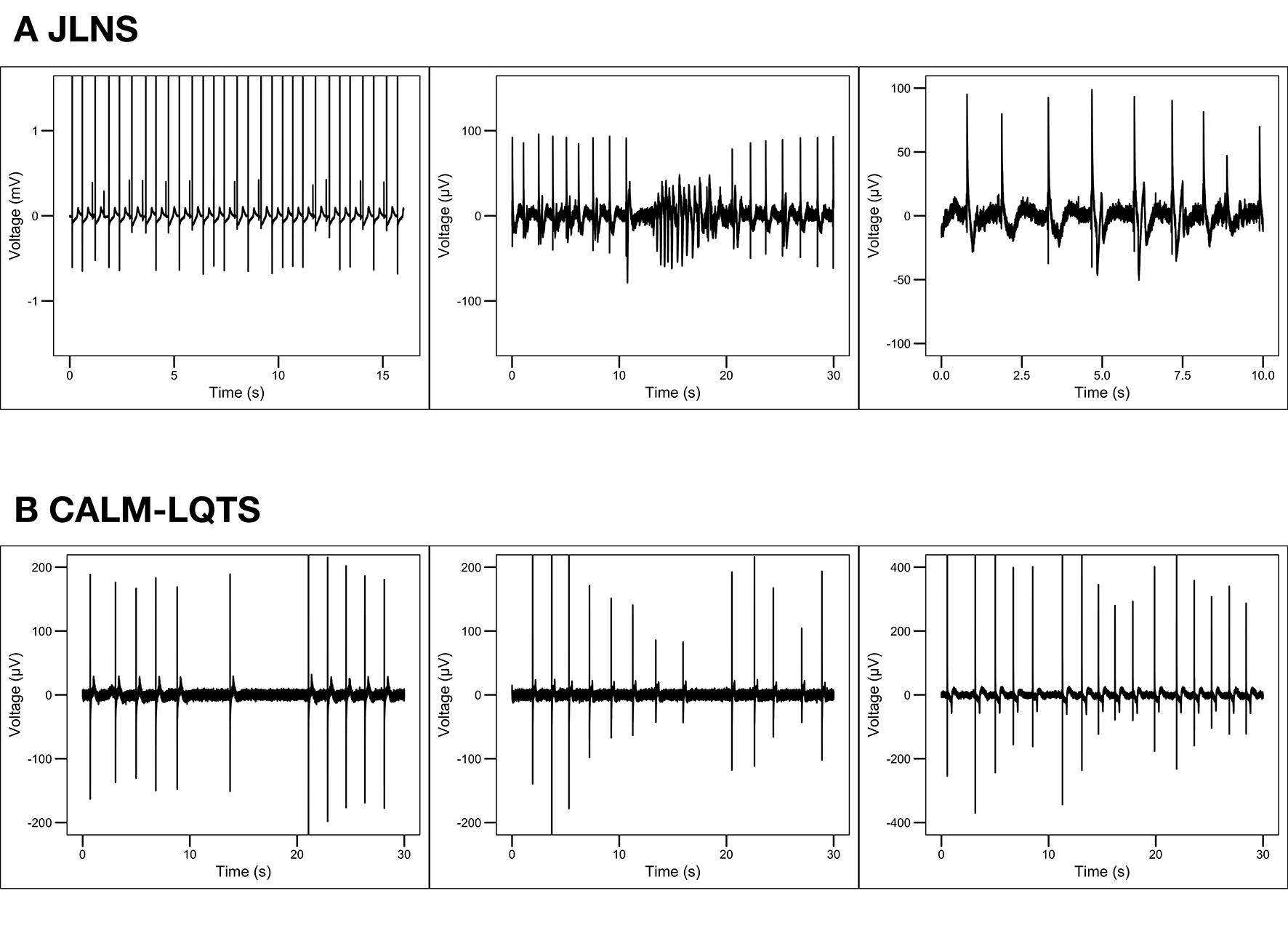

Supplement: Supplementary file 5 [file Image_4.TIFF]
